# Translation from the Ribosome to the Clinic: Implication in Neurological Disorders and New Perspectives from Recent Advances

**DOI:** 10.3390/biom9110680

**Published:** 2019-11-01

**Authors:** Kelvin K. Hui, Yi-Kai Chen, Ryo Endo, Motomasa Tanaka

**Affiliations:** Laboratory for Protein Conformation Diseases, RIKEN Center for Brain Science, Wako, Saitama 351-0198, Japan; kelvin.kw.hui@brain.riken.jp (K.K.H.); yi-kai.chen@riken.jp (Y.-K.C.); ryo-endo@brain.riken.jp (R.E.)

**Keywords:** neurological disorders, phase separation, mRNA translational regulation, ribosome-associated quality control, ribosome stalling, tRNA dynamics

## Abstract

*De novo* protein synthesis by the ribosome and its multitude of co-factors must occur in a tightly regulated manner to ensure that the correct proteins are produced accurately at the right time and, in some cases, also in the proper location. With novel techniques such as ribosome profiling and cryogenic electron microscopy, our understanding of this basic biological process is better than ever and continues to grow. Concurrently, increasing attention is focused on how translational regulation in the brain may be disrupted during the progression of various neurological disorders. In fact, translational dysregulation is now recognized as the *de facto* pathogenic cause for some disorders. Novel mechanisms including ribosome stalling, ribosome-associated quality control, and liquid-liquid phase separation are closely linked to translational regulation, and may thus be involved in the pathogenic process. The relationships between translational dysregulation and neurological disorders, as well as the ways through which we may be able to reverse those detrimental effects, will be examined in this review.

## 1. Introduction

With recent advances in our understanding of translational regulation, we have also uncovered how defects in such regulatory mechanisms contribute to various human pathologies. Protein synthesis is especially critical to the development, survival, and proper functioning of neurons due to their unique cellular architecture which requires specific spatiotemporal regulation. Furthermore, it is well-established that synaptic plasticity, the molecular mechanism underlying learning and memory, requires protein synthesis. Thus, it is not surprising that translational dysregulation has been implicated in a wide spectrum of neurological conditions, ranging from neurodevelopmental to neuropsychiatric to neurodegenerative disorders.

This review begins with an overview of our current knowledge regarding the contribution of translational dysregulation to the pathogenesis of neurological disorders, followed by discussions on recent advances in translational regulatory mechanisms and impending research questions to be addressed, and finally attempt to pinpoint how we may be able to use this newly found knowledge to develop novel strategies for therapeutic treatments.

## 2. Translational Dysregulation in Neurological Disorders

### 2.1. Autism Spectrum Disorders and Other Neurodevelopmental Deficits

**Figure 1:**
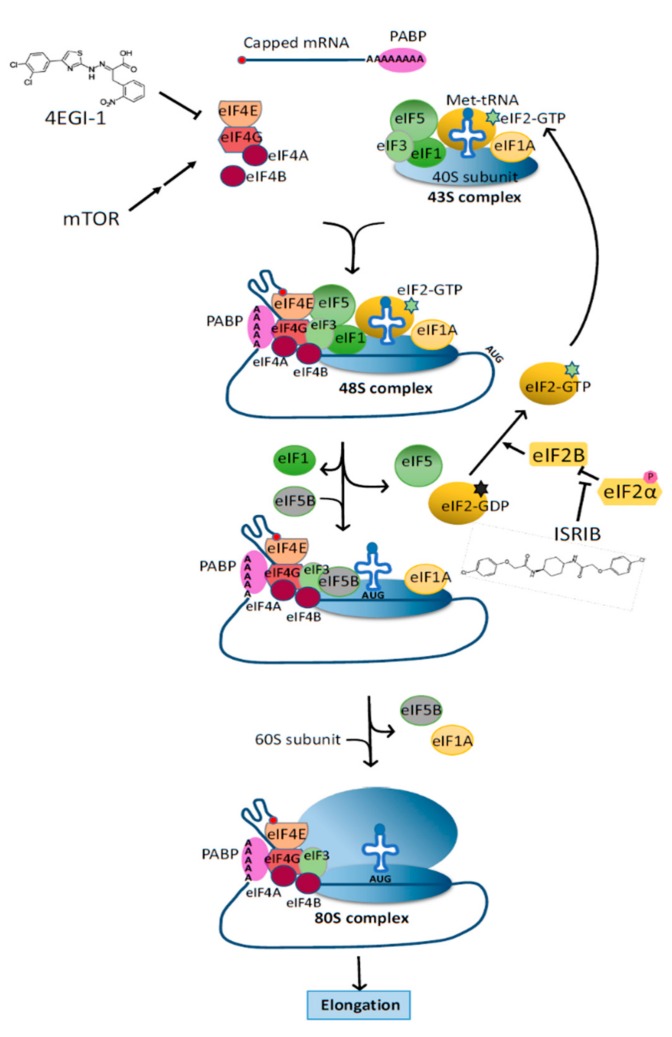
Regulation of Cap-dependent translation initiation. Translation begins with the recruitment of the preinitiation complex (40S ribosome, eIF1, eIF1A, eIF3, eIF5, and the eIF2-GTP-Met-tRNA_i_ complex) to the 5′cap of mRNAs by the eIF4F complex (eIF4A eIF4E, and eIF4G) and eIF4B. This preinitiation complex scans the mRNA for a start codon (AUG) in a 5′ to 3′ manner. Upon recognition of the start codon, eIF5B mediates the release of initiation factors eIF1, eIF2-GDP, and eIF5, allowing the 60S ribosome to join and form the elongation complex 80S ribosome. eIF2B facilitates the recycling of eIF2-GDP to eIF2-GTP, but is inhibited by the phosphorylated form of eIF2α. In many monogenic forms of syndromic autism, mTOR hyperactivation occurs as a result of disturbances in upstream signalling pathways, and in turn enhances translation initiation by direct or indirect phosphorylation of 4E-BP, eIF4B, and eIF4G. A pharmacologic inhibitor of the interaction between eIF4E and eIF4G (4EGI-1) has been shown to have therapeutic benefits for multiple ASD models that have increase translation translational dysregulation. Conversely, a small compound known as ISRIB (integrated stress response inhibitor) can nullify the inhibitory effects of phosphorylated eIF2α on eIF2B [20]. Although it was observed to enhance spatial and fear-associated learning in mice and rats, it remains to be seen whether it can also prevent the decline in cognitive functions in AD and other neurodegenerative diseases.

Much of our current knowledge on autism spectrum disorder (ASD) is derived from work on animal models of syndromic forms of ASD, which are monogenic disorders caused by mutations in genes including *FMR1* (fragile X syndrome), *TSC1*/*TSC2* (tuberous sclerosis), *MECP2* (Rett Syndrome), *UBE3A* (Angelman syndrome), and *SHANK3* (Phelan-McDermid syndrome) (reviewed by [1]). Deficits in translational regulation have been identified in several of these disorders, and largely impinge upon mTOR-regulated translation initiation (Figure 1).

Importantly, both genetic and pharmacologic manipulation of translational regulators have been demonstrated in those animal models to correct for at least some of the observed ASD-like abnormalities [2,3,4,5,6,7,8]. Due to its proposed role as a translation repressor, numerous studies have focussed upon the mRNAs bound by FMRP, which are thought to be dysregulated in its absence [9,10]. Consistent with its association in ASD pathogenesis, many FMRP target mRNAs encode for synaptic proteins and neurotransmitter receptors, with some of them being ASD-associated genes themselves. However, more recent studies have also offered a contrasting view and put into question whether FMRP really represses the translation of its target mRNAs and suggest that at least some of the differentially translating mRNAs in *Fmr1* KO models are compensatory adaptations [11,12,13]. While the precise defects on translational regulation caused by the loss of FMRP functions remain to be elucidated, there is little doubt that translational dysregulation makes a significant contribution to FXS pathology. Aside from FMRP and TSC1/2, additional translational regulators in which mutations have been identified in ASD patients include CYFIP1 [14], EIF4E [15], EIF3G [16,17], and EEF1A2 [16,18,19].

In addition to the above studies regarding defective translational regulators, a recent study had identified the impairment of amino acid transport across the blood brain barrier (BBB) as a cause of ASD [21]. The group further found that mice deficient of the large neutral amino acid transporter 1 (LAT1, encoded by the *SLC7A5* gene) in the BBB endothelial cells, where it is predominantly expressed, showed significant alterations in gene expression in the brain by transcriptome analysis and ASD-like behavioural abnormalities. Specifically, the amino acid response pathway, induced by amino acid deprivation, was activated. Furthermore, 4EBP1 expression and eIF2α phosphorylation were increased, consistent with a reduction of translation efficiency detected by polysome profiling of the mutant mice. This work and the previous observation that the loss of branched chain ketoacid dehydrogenase kinase (BCKDK) [22] is associated with ASD together point to amino acid availability in the brain as a causal pathogenic mechanism. In both studies, the supplementation of branched-chain amino acids (BCAAs), either through a BCAA-enriched diet or direct intracerebroventricular (ICV) injections of leucine and isoleucine, were able to reverse the abnormal behaviours in mice, thus pinpointing amino acid deficiency as the mechanism responsible for the ASD-like abnormalities and further reinforces the idea that translational dysregulation is central to the ASD pathogenic process.

### 2.2. Neuropsychiatric and Mood Disorders

Although not as extensively studied as a causal pathogenic factor as for neurodevelopmental disorders, recent studies have also begun to identify alterations in translational control in neuropsychiatric and mood disorders. For example, a recent study found increased expression of translational machinery in neural progenitor cells (NSCs) derived from induced pluripotent stem (iPS) cells collected from schizophrenic patients compared to matched healthy controls [23]. Furthermore, among the genetic factors which were identified by genome-wide association studies (GWAS) to be linked to schizophrenia, the 15q11.2 copy number variant (CNV) appears to be a prominent risk factor among various neuropsychiatric disorders [24,25]. CYFIP1 is one of the genes commonly deleted as part of the 15q11.2 CNV and is known to form mutually exclusive protein complexes with FMRP/eIF4E and the Wave Regulatory Complex (WRC) to regulate cap-dependent mRNA translation and actin cytoskeletal dynamics, respectively [26,27]. CYFIP2, a lesser known paralogue of CYFIP1 located on chromosome 5, was identified as a major genetic determinant for binge eating behaviour, a form of obsessive-compulsive behaviour [28]. Due to the dual functions of CYFIPs, it remains unclear which is more critical to the manifestation of abnormal neuropsychiatric behaviours.

In addition, several recent studies have highlighted the importance of proper translational control in regulating behaviours associated with neuropsychiatric and mood disorders [29,30,31]. Trinh and colleagues [30] demonstrated that the disruption of translational regulation by a brain-specific deficiency of PKR-like ER kinase (PERK) resulted in impaired behavioural flexibility among other behavioural deficits. Notably, the authors found that mRNA translation of ATF4 regulated by eIF2α to be critical for behavioural flexibility and that PERK and ATF4 expression are reduced in schizophrenic patients. Moreover, it was found that the enhancement of NMDAR function restored downstream eIF2α phosphorylation, ATF4 expression, and behavioural flexibility, thereby signifying that positive modulation of NMDAR signalling is involved and could be a potential therapeutic target.

Conversely, Aguilar-Valles and colleagues [31] showed that the inhibition of eIF4E phosphorylation by genetic and pharmacologic means led to serotonergic dysfunction and depression-like behaviours as a result of reduced translation initiation of IκBα, a negative regulator of NF-κB and pro-inflammatory response, which consequently led to enhanced TNFα production. In addition, the behavioural abnormalities were rescued when the authors blunted the brain inflammation by administering a dominant negative mutant of TNFα. Together, these findings suggest that the translational dysregulation of pro-inflammatory genes may disrupt neuronal functions and behaviour, consistent with the putative role of glial cells in modulating neuronal function by mediating synaptic pruning or regulation of neurotransmitter turnover and metabolism (reviewed by [32,33]).

Finally, a recent study identified Disrupted in Schizophrenia 1 (DISC1) to be critical to the translation initiation of postsynaptic proteins and could be responsible for some of the neuropsychiatric symptoms displayed by patients with frontotemporal dementia (FTD) [29]. In the study, it was observed from brain samples of FTD patients that DISC1 was co-aggregated with the causal protein TDP-43, and that DISC1 normally facilitates the activity-dependent translation initiation of postsynaptic proteins. The co-aggregation event compromised this function of DISC1, and in turn led to reduced expression of numerous postsynaptic proteins, hyperactivity and disturbed sociability in mice with TDP-43 aggregation. Notably, these deficits were reversed when functional DISC1 protein was supplemented, thus demonstrating that DISC1-mediated translational regulation is critical to synaptic functions and contributes to the manifestation of neuropsychiatric symptoms when disrupted. Together, these few examples demonstrate the importance of precise translational regulation to proper neuronal functions related to neuropsychiatric and mood disorders. Further studies are needed in order to understand how other disease-associated genes may impact translational regulation in the brain.

### 2.3. Neurodegenerative Disorders

**Figure 2:**
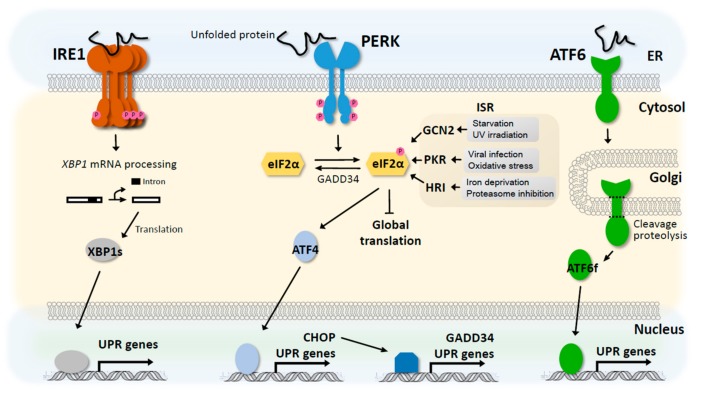
ER stress, unfolded protein response, and the integrated stress response. Multiple pathways, collectively known as the unfolded protein response, are activated by the detection of misfolded proteins in the ER. This ER stress can be sensed by ATF6, PERK, and IRE1, which act via distinct mechanisms to help alleviate the stress by increasing the protein folding capacity of the ER or decreasing the ER protein folding load. Whereas ATF6 and IRE1 mediate a direct and indirect (via mRNA splicing) transcriptional response, respectively; PERK acts to reduce global protein synthesis by enhancing eIF2α phosphorylation. While global translation is reduced by phosphorylated eIF2α, the translation of a small number of transcripts including ATF4 are preferentially induced, which in turn transcriptionally activate genes to promote survival under stress conditions or induce apoptosis. Together with PERK, GCN2, PKR, and HRI are three other kinases known to phosphorylate eIF2α at serine 51 in response to different types of stress, forming the integrated stress response. Aside from HRI, which is not highly expressed in the brain, the ISR kinases have been shown to be activated in various neurodegenerative diseases and may contribute to the pathology by chronically depressing global protein synthesis.

Neurodegenerative diseases including Alzheimer’s Disease (AD), Parkinson’s Disease (PD), and Huntington’s Disease (HD) are caused by the misfolding and aggregation of causal proteins such as amyloid beta, alpha synuclein, and huntingtin, respectively. The formation and accumulation of protein aggregates in affected neurons result in the overactivation of various cytoprotective mechanisms such as the unfolded protein response (UPR). The UPR comprises of three signalling pathways involving PERK, inositol-requiring transmembrane kinase/endoribonuclease 1α (IRE1α), and activating transcription factor (ATF6). Whereas the IRE1α and ATF6 pathways largely result in the transcriptional activation of UPR genes including chaperones, redox enzymes, and ER-associated degradation (ERAD) proteins, PERK phosphorylates eIF2α to suppress cap-dependent translation via the inhibition of eIF2B activity (Figure 2). In terms of the relationship between eIF2α activation and neurodegeneration, increases in the phosphorylation of PERK and eIF2α have been observed in the brains of AD and PD patients [34,35,36,37]. In addition to PERK, other kinases including double-stranded RNA-activated protein kinase (PKR), general control non-derepressible-2 kinase (GCN2), and heme-regulated inhibitor kinase (HRI), can phosphorylate eIF2α and together make up the integrated stress response (ISR). Enhanced PKR activation has been observed in patients and mouse models of AD, PD, and HD [38,39,40,41,42,43,44,45], and the inhibition of PERK, PKR, and GCN2 via genetic and pharmacologic means have shown beneficial effects [46,47,48,49]. Thus, despite eIF2α and the ISR being a protective mechanism that temporarily halts protein translation in order to alleviate further stress caused by protein misfolding and aggregation, chronic eIF2α-mediated shutdown of global protein synthesis may have substantial negative impact on various neuronal functions that require *de novo* protein production.

In addition to the demonstrated links between abnormal eIF2α-regulated translation and AD, it was recently revealed that a reversal of altered eEF2 signalling previously identified in AD [50] has benefits at the cellular and behavioural level in AD model mice. The phosphorylation of eEF2 at threonine 56 is solely regulated by eEF2 kinase (eEF2K) and suppresses eEF2-mediated translation elongation [51]. In the recent study by Beckelman and colleagues [52], eEF2 hyperphosphorylation was observed in the hippocampi of AD patients and Tg19959 AD model mice. Consistent with the observed increase in eEF2 phosphorylation, a reduction in global protein synthesis was detected in the AD mice. Notably, the authors showed that the genetic reduction of eEF2K partially rescued this deficit in protein translation, and reversed cognitive and long-term potentiation (LTP) deficits of two distinct AD mouse models (Tg19959 and APP/PS1), thus revealing the role of translational dysregulation caused by abnormal eEF2 phosphorylation in AD.

Furthermore, a recent study identified a specific reduction of tRNA synthetases in the cerebellum of AD patients by mass spectrometry [53]. Consistent with dysfunctional ribosomes and impairment in protein synthesis being early events in AD pathogenesis [54], a reduction of tRNA synthetases could directly lead to a reduction of protein synthesis required for learning and memory in AD, but may also exacerbate the pathology via tRNA-induced ribosomal stalling, to be discussed in detail below. Though a reduction of polysomal mRNA translation in AD brains was identified as early as 30 years ago [55], we are now only beginning to understand the molecular basis of that observation. While we have highlighted here mainly a role of abnormal eIF2α phosphorylation and its influence on translation initiation as a source of translational dysregulation in AD, there is emerging evidence that other aspects of the process including translation elongation may be altered in AD [52,56,57,58].

## 3. Translational Stalling and Neurodegeneration

Much of the translational regulatory mechanisms discussed thus far largely concerns how various factors and signalling pathways impinge upon the regulators of translation initiation and elongation. However, several recent studies have begun to reveal the detrimental effects of ribosome stalling and the importance of surveillance pathways which function to deal with stalled ribosomes.

### 3.1. tRNA-Induced Ribosome Stalling

The central dogma of molecular biology is that DNA is transcribed into RNA and RNA is in turn translated into proteins. This information flow ultimately requires the proper decoding of mRNAs by tRNAs to convert information stored in the form of nucleic acid into proteins that can carry out biological functions. Numerous neurodegenerative conditions have been associated with defective tRNA dynamics, as extensively reviewed recently by Kapur and colleagues [59] and will not be discussed in detail here. Instead, the discussion will focus on ribosome stalling and employ tRNA-mediated ribosome stalling as an example of how it impacts the brain.

A recent study identified that the loss of GTP-binding protein 2 (*Gtpbp2*) together with a single nucleotide mutation with the n-Tr20 arginine tRNA gene resulted in massive neurodegeneration [60]. The mutation identified between C57BL/6J and C57BL/6N strains led to significantly reduced levels of a brain-specific tRNA^Arg^_UCU_ (encoded by the *n-Tr20* gene) due to the disruption in its pre-tRNA processing. Remarkably, the authors observed increased ribosome stalling at arginine AGA codons in C57BL/6J brains as indicated by a dramatic increase in ribosome occupancy in their ribosome profiling data, which was further exacerbated by the deletion of *Gtpbp2*. Although the exact function of GTPBP2 remains unclear, its homology to no-go/non-stop mRNA decay protein Hsp70 subfamily B suppressor 1-like (HBS1L) and direct interaction with Pelota, a protein involved in ribosome release [61], indicate that it plays a crucial role in the rescue and recycling of stalled ribosomes. A further analysis of the double mutant mice identified the upregulation of the GCN2-eIF2α-ATF4 pathway in response to increased ribosome stalling [62]. The precise mechanism underlying GCN2 activation in response to ribosome stalling remains to be determined, but was demonstrated by the authors to be independent of an increase in uncharged tRNA. Importantly, GCN2 appears to play a cytoprotective role against the ribosome stalling-induced neurodegeneration as the additional deletion of *Gcn2* worsened the phenotype. Notably, mutations in *GTPBP2* have been identified by subsequent studies in individuals displaying either neurodevelopmental impairments or neurodegeneration [63,64], further implicating abnormal ribosome stalling as a cause of translational dysregulation associated with neurological disorders. Therefore, there is an increasing need to examine tRNA dynamics directly in the brain. Next generation sequencing methods have been devised to quantify changes in tRNA expression and modifications [65,66]. Furthermore, a method based on ribosome capture was developed recently to directly examine the tRNAs being used by translating ribosomes [67]. These and other novel techniques will help to address additional questions about tRNA and ribosome dynamics in neurological disorders.

### 3.2. Novel Pathway of Co-Translational Quality Control and Neurological Disorders

As was noted by Ishimura and colleagues [60], the mutation in *n-Tr20* identified in C57BL/6J mice alone caused a significant increase in ribosome stalling, which normally could be compensated by the functions of GTPBP2 to prevent neurodegeneration. In fact, an avalanche of work originally performed in yeast and more recently in mammalian cells has identified a suite of proteins involved in resolving problems associated with stalled ribosomes, a process aptly named ribosome-associated quality control (RQC) as extensively reviewed recently [68]. Prior to the initiation of RQC, GTPBP2 along with HBS1L and Pelota function to sense stalled 80S ribosomes, which are then split into 40S and 60S subunits by ATP-binding cassette protein subfamily E member 1 (ABCE1). Notably, though HBS1L and Pelota are structurally similar to eERF1 and eERF3, respectively, the splitting event mediated by the HBS1L/Pelota complex leaves the peptidyl-tRNA intact [69]. The associated mRNA is degraded by Xrn1 and the exosome complex to prevent further translation [70,71], with the 40S being recycled for subsequent rounds of translation initiation [72] or rapidly degraded via 18S non-functional rRNA decay (NRD) [73,74,75].

**Figure 3:**
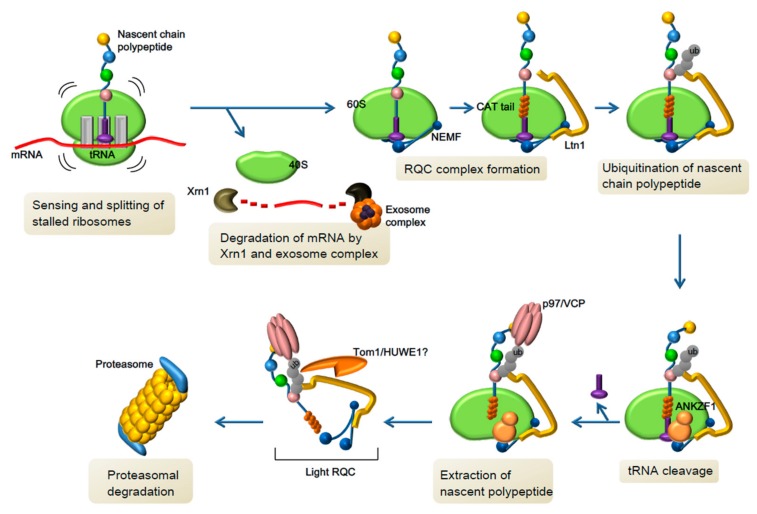
Ribosome-associated quality control. The RQC pathway is initiated after the sensing and splitting of stalled ribosomes by proteins including GTPBP2, HBS1L, Pelota, and ABCE1. Whereas the 40S-associated mRNA is degraded by exonuclease Xrn1 and the exosome complex, RQC mediates the ubiquitination, CATylation, and extraction of the nascent chain polypeptide for its eventual degradation by the proteasome. The RQC complex is mainly consisted of Ltn1 and NEMF, which recognizes aspects of a stalled 60S subunit, including a protruding tRNA and a surface which would otherwise be interacting with the 40S subunit. In this complex with the 60S ribosome, the RING domain of Ltn1 is perfectly situated such that it sits near the ribosome exit tunnel, thus allowing it to ubiquitinate the nascent chain polypeptide. Conversely, NEMF mediates the mRNA- and 40S-independent addition of alanine and threonine residues (CAT tails) to the emerging polypeptide. Finally, NEMF dissociates and is replaced by ANKZF1 to mediate the tRNA cleavage so that the nascent polypeptide chain can be extracted by p97/VCP and its cofactors. The loss of RQC activity due to genetic removal of its principal components have been shown to result in toxicity in yeast and an ENU-induced *Ltn1* mutant was found to cause neurodegeneration in mice. Much remains to be examined to determine how disruptions in the RQC pathway may affect brain functions and whether it has a role in the pathogenic process of various neurological disorders.

RQC in turn is a co-translational quality control pathway aimed at eliminating nascent polypeptide chains remaining on stalled 60S subunits following ribosome splitting [76,77] (Figure 3). Ltn1/listerin binds to the 60S subunit via two distinct domains: the N-terminal domain interacts with the 60S subunit near the interface that normally binds 40S, while the C-terminal domain sits at the ribosome exit tunnel such that the Really Interesting New Gene (RING) finger domain is perfectly situated to ubiquitinate the protruding nascent polypeptide chain [78,79,80]. The cryogenic electron microscopy (cryo-EM) structures also beautifully illustrated how the second component of RQC, nuclear export mediator factor (NEMF), recognizes free 60S subunits dissociated from stalled ribosome and further prevents the re-association with another 40S. NEMF accomplishes this function by interfacing both with a surface composed of ribosomal proteins and rRNA of the 60S and binding to the peptidyl-tRNA exposed on the P site following ribosome splitting. Following their association with the 60S subunit, Ltn1 and NEMF function separately to facilitate the degradation of the nascent polypeptide chain by ubiquitination and CATylation, a mRNA- and 40S-independent polypeptide extension process using alanine and threonine residues, respectively [79,81]. The C-terminal Ala/Thr extensions (CAT tails) were initially believed to help push out and expose lysine residues hidden inside the ribosome exit tunnel for ubiquitination by Ltn1 [81]. A more recent study, however, suggested that CAT tails enhance the ability of Ltn1 to target structured polypeptides for ubiquitination on the 60S ribosome, or potentially by other E3 ligases away from the ribosome if they do not get processed properly by the RQC pathway [82]. While it remains controversial which, or both, of these mechanistic explanations are correct, it should also be noted that CAT tails generated by NEMF are thought to enhance the aggregation potential of nascent polypeptide chains [83,84,85]. CATylation may thus serve a physiologic function by promoting the nascent polypeptide chain to assume an inert aggregated state for other degradative mechanisms (e.g., macroautophagy).

Prior to nascent polypeptide chain extraction from the 60S subunit, the covalently linked tRNA to the most recently incorporated amino acid in the P site must be cleaved by ANKZF1 [86]. Once ubiquitinated and cleaved, the nascent polypeptide chain is recognized by AAA ATPase p97/VCP and its cofactors, then extracted from the 60S subunit for proteasomal degradation [87]. A distinct mechanism mediated by peptidyl-tRNA hydrolase Ptrh1 was also described recently to facilitate the release of non-ubiquitinated nascent polypeptide chains [88]. A light version of RQC, which includes all RQC components but is separated from the 60S, has been postulated to exist prior to proteasomal degradation in order to protect the nascent polypeptide chain from aggregation or other undesirable events [89]. Notably, this light RQC complex also includes the E3 ubiquitin ligase Tom1/HUWE1, which was previously shown to degrade excessive unassembled ribosomal subunits. This leads to the speculation of whether Tom1/HUWE1 may also be responsible for the degradation of 60S subunits once RQC is completed [90,91]. Importantly, *HUWE1* is the principal candidate gene responsible for non-syndromic X-linked intellectual disability caused by microduplication of Xp11.22 [92,93] and patients possessing *HUWE1* variants were found to exhibit severe intellectual disability [94].

Although the fine details of RQC continue to be revealed, there is already evidence that a compromise of RQC activity can result in neurodegeneration. A genome-wide N-ethyl-N-nitrosourea (ENU) mutagenesis screen identified a recessive mutation in *Ltn1* which caused a splicing defect and dramatically reduced both the expression and activity of Ltn1 in the CNS [95]. The *listerin* mutant mice displayed a progressive loss of neuronal and motor functions due to neurodegeneration. Given that CATylated polypeptides accumulate in the absence of Ltn1 and are aggregation-prone [81,83,85], it is unclear whether this neurodegeneration was caused by the aggregation of CATylated polypeptides or the inability to cope with stalled ribosomes effectively, or both. Certainly, there still remains a large knowledge gap between what happens when RQC occurs properly to mediate the degradation of nascent polypeptide chains on stalled ribosomes and what the consequences are when it cannot be completed. Given that components of the RQC pathway are each tasked with distinct functions, the loss of or reduction in activity of different components may result in unique deficits and phenotypes. Notably, *NEMF* variants were recently identified by exome sequencing in patients with intellectual disability [96]. It will be of interest to see whether future genetic studies will identify additional linkages between mutations in RQC components and neurological disorders.

## 4. Toxic RAN Translation Products in Neurodegeneration

Similar to stalled ribosomes, abnormal translation initiation represents another form of translational dysregulation which has also been associated with various neurological disorders. Repeat-associated non-ATG (RAN) translation, initiated by long stretches of tandem tri- to hexanucleotide repeats, was originally identified from spinocerebellar ataxia type 8 (SCA8) and myotonic dystrophy type 1 (DM1) CAG expansion transcripts [97], but have since been detected in other repeat expansion diseases as well (reviewed by [98]). For many of these repeat expansion diseases, especially HD and various forms of spinocerebellar ataxia (SCA) which are caused by expanded CAG repeats, it has long been a mystery whether the principal culprit of neurotoxicity was the protein product with polyglutamine expanded tracts or the mRNA with poly CUG repeats (as reviewed by [99]). However, the surprising finding that the elimination of the initiation AUG on such transcripts still led to protein translation opened up a whole new avenue of research on these disorders and brought together with it many unanswered questions.

It is now understood that the non-canonical translation initiation occurs at near-cognate AUG codons (e.g., CUG, GUG, and UUG) and can produce protein products in both directions on sense and antisense transcripts [100]. Complicating matters further, frameshifting is known to occur with repeat expansion transcripts such that RAN translation products can be produced from all six reading frames [97,100]. These polypeptide products have been shown to behave differently and vary in their potentials to form protein aggregates [101,102,103,104]. The resulting aggregates further disturb proteostasis by disrupting degradative pathways such as ubiquitin proteasomal degradation and macroautophagy [105,106,107,108,109,110] and enhancing ER stress [109,111].

Aside from the disturbance of proteostasis, RAN translation products generated from the *C9ORF72* G_4_C_2_ hexanucleotide repeats have been demonstrated to disrupt two critical protein complexes: the nuclear pore complex [112,113,114,115] and stress granules [115,116,117,118]. Importantly, the two phenomenon appear to be connected in that nucleocytoplasmic factors were found to mislocalize to stress granules [116], while stress granule components have also been found to be deposited into protein aggregates formed by RAN translated products [119]. This is largely because these polypeptides are capable of undergoing liquid-liquid phase separation (LLPS) themselves [117,118,120], or entering existing membraneless organelles such as stress granules and the nuclear pore complex and alter their properties [117,121]. In addition, the *C9ORF72* repeat RNA was also found to promote phase separation directly [122]. LLPS and translation dysregulation will be further discussed in the next section.

Although RAN translation appears to utilize non-canonical initiation sites, it was previously demonstrated using CGG repeats in the 5′UTR of *FMR1* that translation initiation is similar to canonical translation in that it employs a m^7^G cap, eIF4A-dependent mechanism [123]. It remains to be determined whether this is specific for the *FMR1* CGG repeats or is generally applicable to other RAN translation products as well, because eIF2α phosphorylation, which should inhibit cap-dependent translation initiation, has been shown to upregulate RAN translation [124,125]. Consistently, the deletion of *EIF2A* was found to significantly blunt RAN translation [126], in accordance with the initial observation that CUG-/Leu-tRNA initiation requires eIF2α expression [127]. If indeed cellular stress, via the action of eIF2α and perhaps other factors, does enhance RAN translation, it would mean that the disruption of proteostasis by RAN translation products mentioned earlier would initiate in a vicious feed-forward cycle that exacerbates neurotoxicity.

A recent genetic screen to identify modifiers of RAN translation found that the non-essential ribosomal subunit RPS25 to be required for efficient RAN translation [128]. Interestingly, RPS25 is known to play a critical role in internal ribosome entry site (IRES)-mediated translation [129,130,131,132]. Thus, these data would support a model in which RAN translation is favoured by cap-independent mechanisms of translation initiation [124], rather than being initiated in a cap-dependent manner as previously suggested [123,125,133]. Notably, non-AUG translation was identified to be uniquely resistant to various translation elongation inhibitors such as cycloheximide and anisomycin [134]. Thus, it would appear that RAN translation exhibit unique properties that differentiate it from canonical translation, which fortunately may offer ways through which they could be specifically suppressed in pathologic conditions in order to reduce neurotoxicities associated with it.

## 5. Liquid-Liquid Phase Separation and Neurodegeneration

A tremendous wealth of research advances has been made in recent years on liquid-liquid phase separation (LLPS) and membraneless organelles in mediating biological processes. Aside from substantial implications in neurological disorders, LLPS is now known to be involved in the regulation of a wide spectrum of subcellular compartments including but not limited to nucleolus, heterochromatin, nuclear pore complex, stress granules, P-bodies, and centrosomes. While details about them are emerging on a constant basis, they appear to be formed by proteins with prion-like low-complexity intrinsically disordered domains [135], and their assembly/disassembly can be regulated by changes in local concentration [136,137], cellular environment (e.g., pH and salts) [138], post-translational modifications like phosphorylation [139,140] and ubiquitin-like conjugations [141,142], and interactions with RNA or other proteins [137,140,143,144,145]. RNA modifications, in the form of m^6^A methylation, can similarly influence LLPS dynamics by modulating their interactions with m^6^A-binding proteins YTHDF1, YTHDF2, and YTHDF3 [146]. Furthermore, RNA with repeat expansion alone is capable of undergoing LLPS via multivalent base-pairing [147]. The biological implications of LLPS and how they are governed by their biochemical and biophysical properties have been reviewed in length previously [148,149], and thus the focus here will be on how LLPS impacts translational regulation in neurological conditions.

**Figure 4:**
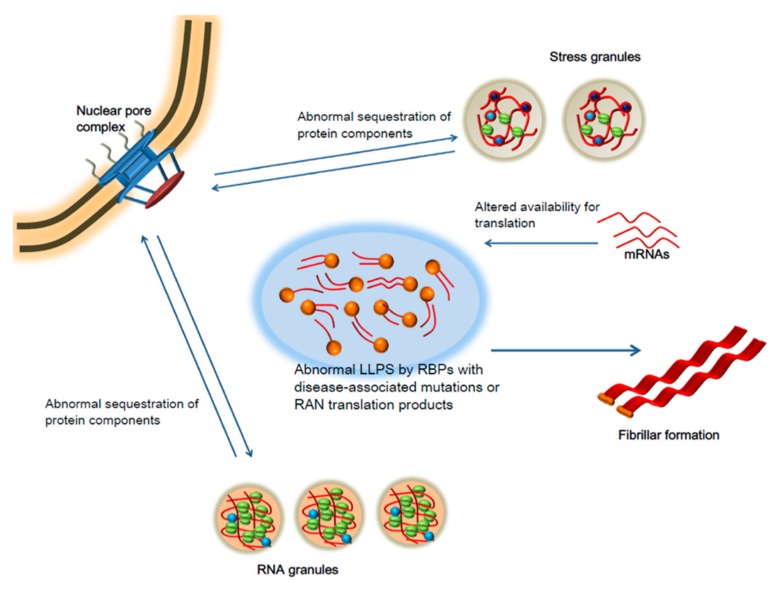
Aberrant LLPS and neurodegeneration. LLPS is now known to play a critical role in a growing number of biological processes and regulates the dynamics of various membraneless organelles in the cell. However, it has been demonstrated recently that aberrant LLPS dynamics caused by either disease-associated mutations of RNA-binding proteins or RAN translation products involved in numerous neurodegenerative diseases contribute significantly to the neurotoxicity via several different mechanisms. LLPS formed by mutant proteins or RAN translation products can: (1) disrupt existing membraneless organelles including the nuclear pore complex, stress and RNA granules; (2) sequester RNAs and proteins which are not normally part of the LLPS assemblies or disturb their exchange dynamics such that their normal functions are disrupted (e.g., mRNA translation may be decreased by the reduced availability of certain transcripts or translation factors); and (3) LLPS assemblies can further undergo conformational changes and ultimately lead to the formation of fibrillar aggregates, further disturbing proteostasis in affected neurons.

LLPS is tightly linked to translational control due to their involvement in the regulation of stress and RNA granules, which function to protect, transport, and regulate mRNA and other RNA species such as long noncoding RNA (lncRNA), micro RNA (miRNA), and tRNAs, to control translation by ribosomes under various cellular conditions (Figure 4). Furthermore, the dynamic nature of these membraneless organelles has been shown to be greatly affected by disease-associated mutations and can significantly contribute to the pathogenic process [145,150,151,152,153]. Multiple RNA-binding proteins (RBPs) linked with neurodegenerative diseases, including FUS, TDP- 43, Ataxin-2, TIA-1, hnRNPA1, and hnRNPA2, are known to undergo LLPS, which is critical to the normal physiological functions of these proteins. However, in most cases disease-associated mutations disrupt LLPS dynamics, causing both an irreversible sequestration of proteins and RNA species [154,155,156] and the conversion to fibrillar aggregates which create additional problems by disturbing proteostasis [136,152,157,158,159,160,161]. López-Erauskin and colleagues [162] recently demonstrated that axonal protein synthesis and synaptic functions are inhibited by FUS mutations associated with ALS/FTD through a gain-of-toxicity mechanism [163,164]. Though not directly observed in the study, the FUS mutants that caused translational dysregulation were previously shown to undergo LLPS [165] and thus could have directly contributed to the observed translational changes. Indeed, other studies have observed reduced protein synthesis due to the selective trapping of translational regulators and RBPs into LLPS assemblies formed by disease-associated FUS mutants and FMRP [155,166,167]. Furthermore, not only may LLPS assemblies sequester critical components needed for proper translational control, mutations could also alter the physical properties of stress and/or RNA granules and their transport dynamics, thus further exacerbating translational dysregulation by disrupting mRNA trafficking and local translation [168].

While it is quite clear that changes to LLPS dynamics caused by disease-associated mutations of various RBPs are crucial to the pathogenic process, it remains to be determined what the contribution is by translational dysregulation given the multiple ways that LLPS assemblies can be damaging to affected neurons. There is still much to be learned about LLPS assemblies. For example, the characterization of proteins and RNAs which are recruited into them and how that may impact neuronal functions will be extremely informative. Importantly, it will be interesting to differentiate between the entrapped proteins and RNAs for each RBPs that can undergo LLPS. Given the dynamic nature of LLPS assemblies, it may be critical to employ novel techniques to label proteins and RNAs in situ as it is quite possible that such assemblies cannot be preserved completely during biochemical procedures like fractionation and immunoprecipitation. Various adaptions of UV crosslinking and immunoprecipitation (CLIP), as reviewed by Lee and Ule [169], have been developed to study various RNA-protein interactions. More recently developed proximity labelling techniques such as APEX2/APEX-Seq [170,171,172,173] and BioID2 [174] may prove to be superior methods and was recently utilized to generate a systematic map of various protein components of stress granules and other mRNA-associated granules [173,175,176]. Direct light-mediated manipulation of stress and RNA granules will also be useful for examining how their dynamics affect translational events and vice versa [136,177].

## 6. Future Perspectives

For a long time, transcriptional control at the mRNA level was believed to be the principle mode of regulation for protein expression. Recent advances in cell biology have changed this view as a multitude of post-transcriptional mechanisms have been discovered. For neurons, with unique spatiotemporal properties unlike any other cells in the body, there are additional needs to regulate protein synthesis in a dynamic fashion that stretches from seconds to days and micrometers to centimeters. In learning about how translational regulatory mechanisms are disrupted in various neuropathologies, we have also gained considerable knowledge on how the brain works. Now, we may be at a point in which we could begin to utilize that knowledge and devise new ways to treat the different types of neurological disorders discussed earlier.

### Promises and Cautions for Novel Therapeutic Strategies

It is now clear that defects in mediating activity-dependent synaptic changes are at the core of multiple neurodevelopmental, neuropsychiatric, and neurodegenerative disorders. Not surprisingly, given its importance in facilitating synaptic plasticity, *de novo* protein synthesis is critically essential and our understanding of the proteins that need to be upregulated and downregulated in a timely manner due to neuronal activity is starting to emerge. Recently, 4EGI-1, a compound that binds to eIF4E and prevents its interaction with eIF4G, was able to rescue synaptic and behavioural abnormalities in several ASD model mice [3,4,6,7]. In addition, genetic rescue of FXS pathology have been shown via the deletion of translational activators such as *S6k1* and *Cpeb* [8,178]. However, caution must be taken when interpreting these successes. A recent study found that the pharmacologic enhancement of muscarinic acetylcholine receptor 4 (M4) has beneficial effects on the pathological changes observed in FXS model mice, despite translatome analysis revealing that the receptor was upregulated in the brains of those animals [12]. Furthermore, rather than increased global protein synthesis due to mTOR hyperactivation, *Tsc2* mutant mice were found to have reduced translation rates and, perhaps even more surprising, shown rescue effects when crossed with *Fmr1* KO mice [2]. Altogether, these studies point to the potential use of pharmacologic modulators of translation regulation in the treatment of ASD and other neurodevelopmental disorders, but a comprehensive understanding of which specific disorders such therapies are applicable for is required.

Another example of a potential therapeutic target is eIF2α due to its aberrant activation in a number of different neurological disorders in which proteostasis is disrupted and in turn suppresses cap-dependent mRNA translation, thereby hampering synaptic plasticity and other neuronal functions which require *de novo* protein synthesis. Furthermore, eIF2α is believed to promote RAN translation in repeat expansion diseases [124,125], thus targeting eIF2α is expected to have dual benefits in that the unwanted brake on cap-dependent translation would be released and that the generation of damaging RAN translation products could be minimized. Cheng and colleagues [124] demonstrated precisely this by inhibiting PERK and the downstream signalling events of phospho-eIF2α by using pharmacologic inhibitors GSK260641 and ISRIB, respectively, in a cellular model of the hexanucleotide expansion of *C9ORF72*. It remains to be seen whether these compounds can ultimately exert cytoprotective effects and show in vivo therapeutic efficacy against diseases with abnormal eIF2α activation.

Aside from the more traditional pharmacologic modulation to correct for translational dysregulation in neurological disorders, there have also been advances using more novel approaches. As in many other areas of biomedical research, there are concurrent efforts to evaluate the use of clustered regularly interspaced short palindromic repeats (CRISPR)/Cas9 to contract the repeat expansions [179] or downregulate repeat expansion transcripts at either the DNA or RNA level [180,181]. Other innovative approaches aimed at preventing the detrimental effects on translational regulation caused by pathologic LLPS assemblies include blocking the nuclear export of repeat expansion transcripts such that RAN translation products cannot be generated [182] and preventing pathologic LLPS of disease-associated RBPs by using nontoxic short repeat RNA [183], bait oligonucleotides [184], or reducing the levels of poly(ADP-ribose) [185]. Continued efforts to assess these and other therapeutic approaches to correct for translational dysregulation associated with various neurological disorders provide new glimpses of hope for treating such devastating diseases.

In conclusion, novel therapeutic strategies based on our better understanding of translational regulatory mechanisms are emerging and show promise in reversing many of the deficits in cellular and/or animal models of various neurological disorders. With continued growth in this knowledge, we can anticipate further advances in these areas, which will hopefully be translated to clinical use for patients in the near future.
